# ABCC8 Mutation Causing Permanent Neonatal Diabetes Mellitus in Early Infancy: A Case Report

**DOI:** 10.1055/a-2667-6711

**Published:** 2025-08-12

**Authors:** Leul M. Manyazewal, Mikiyas G. Teferi, Helina K. Teklehaimanot, Michael A. Negussie, Leleul M. Demeke, Absira B. Abate

**Affiliations:** 1 School of Medicine, Department of Pediatrics and Child Health, College of Health Sciences, Addis Ababa University, Addis Ababa, Ethiopia

**Keywords:** neonatal diabetes mellitus, ABCC8 mutation, sulfonylurea, genetic diagnosis, low-resource settings, case report

## Abstract

**Introduction:**

Neonatal diabetes mellitus (NDM) is a rare monogenic form of diabetes presenting within the first 6 months of life. It can be transient or permanent; early diagnosis is essential to improve outcomes.

**Case Presentation:**

A 45-day-old male infant presented with fever, dehydration, and marked hyperglycemia. Initially misdiagnosed as meningitis, further evaluation revealed diabetic ketoacidosis, confirmed by elevated blood glucose and +4 urine ketones. He was stabilized with IV fluids and insulin, then transitioned to subcutaneous insulin. Persistent hyperglycemia and patient's age raised suspicion for NDM, warranting genetic testing, which identified a heterozygous pathogenic ABCC8 missense variant. Oral sulfonylurea was initiated using a locally compounded suspension due to limited resources. Insulin was successfully tapered, and euglycemia was achieved on sulfonylurea monotherapy.

**Discussion:**

Highlighted here is the importance of genetic testing in suspected NDM; it directly guides management. Shifting from insulin to oral agents improves glycemic control and long-term prognosis. Managing NDM in low-resource settings requires adaptive, multidisciplinary approaches. Ideally, patients should be followed into adolescence, focusing on neurodevelopment, as some variants may lead to neurological complications.

**Conclusion:**

Recognizing NDM in infants with unexplained hyperglycemia is important for timely, targeted treatment. Individualized care is possible in constrained settings, offering improved overall outcome.

Neonatal diabetes mellitus (NDM) is a rare but serious form of diabetes that presents within the first 6 months of life. The global incidence of NDM is relatively small, estimated to be between 1 in 90,000 to 1 in 160,000 live births. 1 In contrast to the more prevalent autoimmune-mediated Type 1 diabetes mellitus, NDM is nonautoimmune, caused by monogenic mutations. 1 2 Early recognition of this distinction is critical, as the underlying genetic etiology directly influences both the clinical management and long-term prognosis of affected infants. 2 3

NDM is broadly classified into two types: transient neonatal diabetes mellitus (TNDM) and permanent neonatal diabetes mellitus (PNDM). While both types may present with hyperglycemia in the first few weeks of life, TNDM often resolves spontaneously within the first year of life. PNDM, however, persists indefinitely and often requires lifelong treatment. 1 4 5 Numerous studies have identified specific mutations in multiple genes that are thought to be directly linked with the pathogenesis of NDM. Mutations in KCNJ11, INS, and ABCC8 are among the most commonly implicated. 4 6

The significance of this clinical encounter lies not only in highlighting the importance of early diagnosis but also in demonstrating that a personalized approach to treatment will eventually lead to better outcomes. 4 In this patient, identification of a pathogenic ABCC8 missense variant allowed for a timely shift from conventional insulin therapy to oral sulfonylurea (glibenclamide), a medication that directly targets the defective K-ATP channel, which has been demonstrated on several similar cases to restore insulin secretion in patients with these specific mutations. 3 6

We also explore the growing importance of maintaining a high degree of vigilance to recognize when to use molecular diagnostics techniques in routine pediatric care, even in low-resource health care systems. 5 7 Genetic diagnosis can spare patients with NDM from the lifelong burden of insulin therapy while also offering a safer, easier, and more affordable oral treatment option, ultimately enhancing both the quality of life and long-term prognosis of these patients. 2 3 4

Here, we present the case of an infant diagnosed with PNDM due to a pathogenic ABCC8 mutation, focusing on the diagnostic journey and the unique and successful therapeutic transition within a low-resource setting.

## Case Presentation

A 45-day-old male infant presented to the pediatric emergency ward with a 1-day history of fever, poor feeding, and irritability, which began 2 days after receiving routine immunization. The mother also reported that he had sunken eyes and was crying without tears. On examination, the infant had a fever of 38.0°C, tachypnea of 55 breaths per minute, tachycardia of 145 beats per minute, and oxygen saturation of 94%.

The infant appeared clinically dehydrated, with sunken fontanelle, dry oral mucosa, and capillary refill of 3 seconds. He was alert but irritable, with no dysmorphic features. Cardiovascular and respiratory examinations were unremarkable. On neurological examination, there was a normal tone and intact primitive reflexes, with no focal deficits or seizure activities observed. There was a normal external male genitalia on the genitourinary exam. A presumptive diagnosis of early-onset neonatal sepsis with meningitis was initially made and empirical IV antibiotics and fluid resuscitation were initiated.

Laboratory results showed blood culture and cerebrospinal fluid (CSF) analysis were both unremarkable. However, there was significant hyperglycemia with the random blood sugar ranging from 468 to 512 mg/dL, and urinalysis was strongly positive for ketones (+4 initially). The electrolyte panel showed mild hyperkalemia and hyponatremia ( Table 1 ). Subsequently, the diagnosis was revised to diabetic ketoacidosis (DKA).

**Table 1:** Key laboratory findings at admission and subsequent days

Parameter	At admission	After resuscitation	After insulin initiation	Reference range
Blood glucose (random)	653 mg/dL	580 mg/dL	110–250 mg/dL	45–125 mg/dL (neonate)
Ketones (urine)	Positive (++ + +)	Positive (++ + )	Negative	Negative
Arterial blood gas (ABG)	pH 7.31, HCO _3_^−^ 16 mmol/L	7.35 19 mmol/L	7.36 21 mmol/L	pH 7.35–7.45, HCO _3_ 22–26 mmol/L
Electrolytes Na ^+^ K+	129 mmol/L 6.2 mmol/L	136 mmol/L 5.36 mmol/L	– 4.8 mmol/L	Na ^+^ 135–145 mmol/L K+ 3.5–5.5 mmol/L
Serum creatinine	0.3 mg/dL	0.2 mg/dL	–	0.3–1.0 mg/dL
Thyroid function tests (TSH, fT4)	TSH—3.2 mIU/L fT4—1.7 ng/dL	–	–	Age-appropriate ranges TSH—0.6–7.0 mIU/L fT4—1.0–2.5 ng/dL

Management of DKA was initiated according to the Ethiopian Clinical Reference Manual for Advanced Neonatal Care (2021). 8 The patient continued to receive IV fluid resuscitation with isotonic saline to address moderate dehydration, followed by maintenance fluid therapy until he was able to resume breastfeeding. Continuous IV regular insulin was initiated at a dose of 0.05 units/kg/h via infusion and titrated as needed. Once the blood glucose level decreased below 250 mg/dL and urine ketones cleared, the IV insulin was gradually tapered down and eventually transitioned to a subcutaneous regimen consisting of Neutral Protamine Hagedron (NPH) and postprandial regular insulin.

Despite clinical stabilization, euglycemia could not be maintained, with blood glucose levels fluctuating between 200 and 350 mg/dL, even though urine ketones became negative and electrolytes improved. The treating team decided to conduct genetic testing considering the persistent hyperglycemia and the age of the patient.

Genetic testing revealed a heterozygous missense variant in the *ABCC8* gene: NM_001287174.1:c.1760T > G p.(Val587Gly), located at Chr11:g.17452418 (GRCh37) ( Table 2 ). This variant has been previously reported in association with neonatal diabetes and is classified as pathogenic, as described by Babenko et al and Rafiq et al. 6 7 Classification followed the American College of Medical Genetics guidelines, which integrates clinical, functional, and population data to determine pathogenicity. 9

**Table 2:** Genetic testing, diagnosis, and characteristics

Categories	Details
Gene involved	*ABCC8*
Variant type	Missense mutation (activating)
HGVS notation	c.1760T > G; p.(Val587Gly)
Genomic location	Chr11:g.17452418 (GRCh37)
Zygosity	Heterozygous
Inheritance	De Novo
Classification	Pathogenic
Reason for testing	Diagnostic workup for persistent neonatal hyperglycemia
Methodology	Sanger sequencing of *ABCC8* , *KCNJ11* , and *INS* genes
Familial risk	50% risk of transmission to offspring

Note: Narrative summary—genetic testing confirmed a heterozygous de novo pathogenic missense mutation in the *ABCC8* gene (c.1760T > G; p.Val587Gly), associated with neonatal diabetes.

Subsequently, diagnosis of persistent NDM was considered, and it was decided that, instead of insulin, the patient would benefit more from oral sulfonylureas, such as glibenclamide. However, the ideal suspension formulation was not available commercially. As an alternative, the oral tablets were carefully crushed into fine powder and reconstituted into a predetermined volume of sterile distilled water.

Five milligrams glibenclamide tablets were powdered at the hospital pharmacy and dissolved in 5 mL of sterile distilled water to create a consistent suspension. Doses were measured using a 3-mL syringe to ensure precision and administered orally. Treatment was initiated at 0.1 mg/kg/d, divided into morning and evening doses. Blood glucose was closely monitored before each dose and around feeding times. There were no episodes of hypoglycemia. The dose was gradually increased, and stable euglycemia was achieved at 1 mg/kg/d. Insulin was gradually tapered and eventually discontinued. The patient exhibited no adverse effects to the treatment initiated.

There was no known family history of diabetes mellitus or other related genetic disorders. The infant was the first child, and both parents were nondiabetic. Genetic testing for the infant was made possible through sponsorship and was performed at a laboratory outside the country. Unfortunately, due to financial constraints and the lack of access to testing facilities within the country, genetic testing for the parents was not feasible. Following the diagnosis, the parents were provided with basic genetic counseling. They were informed of the 50% recurrence risk associated with heterozygous pathogenic variants in *ABCC8* , and advised to consider prenatal or early postnatal testing in future pregnancies, should it become accessible. They were also trained in glucose monitoring, as well as the preparation and administration of the glibenclamide suspension.

At discharge, the infant showed significant improvement in clinical condition and laboratory parameters. Neurologically, he was alert and responsive, with age-appropriate tone, intact primitive reflexes, and normal developmental behavior for his age. As part of the discharge workup, abdominal ultrasound and echocardiography were performed and found to be normal.

Long-term follow-up at our hospital was not possible, given the family's residence in a remote part of the country far from the city. Instead, they were referred to a regional primary hospital with pediatric service. A detailed summary of disease course and treatment, including details on the preparation of the compounded glibenclamide suspension, was provided to the local care team, along with protocols for dose adjustments and contact information if further consultation was needed. Regular follow-up every 3 months was recommended, as well as pediatric neurodevelopmental assessment at 6 and 12 months. Additionally, they were encouraged to return in case of treatment failure or emergence of complications requiring specialized care.

## Discussion

PNDM is a rare monogenic type of diabetes that arises during the first 6 months of life and requires lifelong care. 1 It is most frequently caused by activating mutations in genes that encode ATP-sensitive potassium (K-ATP) channel subunits, notably KCNJ11 and ABCC8, which control the release of insulin from β-cells in the pancreas. 1 6 10 NDM has a low prevalence, estimated at 1 in 90,000 to 1 in 160,000 live births, 1 but because of its nonspecific presentation and overlap with more commonly occurring pediatric illnesses, it presents a substantial diagnostic difficulty, especially in low-resource settings.

A key but unnoticed clinical pearl in PNDM is that it may initially mimic common infectious or metabolic illnesses in infancy, leading to diagnostic delays. 5 The infant's manifestation of fever, irritability, poor feeding, and hyperglycemia shortly after routine vaccination raised initial concerns for meningitis, possibly sepsis, especially in the setting of altered clinical appearance and moderate dehydration. However, the laboratory findings of marked hyperglycemia (>500 mg/dL) and ketonuria called for a revision of the initial diagnosis, leading to identification of DKA—a rare but potentially fatal manifestation in infants. 4 5

Initially high on the list of differentials, meningitis and sepsis were ruled out after and negative CSF analysis and blood culture were obtained. Despite being more common overall, type 1 diabetes is exceedingly uncommon before the age of 6 months and is usually autoimmune in nature. 4 6 Additionally, taken into consideration was TNDM, which can resolve on its own within the first year of life. 1 11 However, ketoacidosis is a rare symptom of TNDM, which usually goes away in a matter of weeks to months. 5 11 In contrast, our patient did not show any evidence of remission and remained chronically hyperglycemic despite insulin therapy, making TNDM less likely. Lack of improvement raised concerns for the need for genetic diagnosis. A heterozygous pathogenic missense activating mutation in the *ABCC8* gene was finally verified by genetic testing, conclusively confirming the diagnosis of PNDM, 2 10 which offered a clear therapeutic pathway.

The *ABCC8* gene encodes the sulfonylurea receptor (SUR1) subunit of the ATP-sensitive potassium (K-ATP) channel, a critical component of glucose-stimulated insulin secretion. 6 11 The pathogenic mutations in this gene, specifically activating mutations, have been shown to disrupt normal channel function, impairing glucose-stimulated insulin release, ultimately leading to persistent hyperglycemia starting from early infancy. 4 6

Another frequent cause is an activating mutation in *KCNJ11* , which encodes the Kir6.2 subunit of the K-ATP channel. 6 Like the ABCC8, a mutation in this gene impairs glucose-stimulated insulin secretion. 4 7 Less commonly, mutations in genes such as *INS* —which involves proinsulin misfolding and β-cell stress 1 — *GCK* , *EIF2AK3* (associated with Wolcott–Rallison syndrome), *FOXP3* (IPEX syndrome), and *GLIS3* have been identified, sometimes in association with extrapancreatic features. 5

However, it has been demonstrated repeatedly that the effects of this defect can be pharmacologically reversed by sulfonylureas. 6 10 Sulfonylureas overcome the underlying defect in KATP channel–related PNDM by binding directly to the SUR1 subunit of the ATP-sensitive potassium channel, leading to its closure independently of intracellular ATP levels. 6 10 This action restores membrane depolarization and calcium-mediated insulin release in pancreatic β-cells. It is important to note that the mechanism of action of sulfonylureas in PNDM differs from that in type 2 diabetes. Sulfonylureas augment glucose-dependent insulin secretion in type 2 diabetes while they directly depolarize persistently hyperpolarized β-cells in PNDM thereby, enabling insulin release even in the absence of normal glucose responsiveness. 6 12 Because of this, ideal therapeutic response typically requires doses 2 to 4 times higher than those used in type 2 diabetes. 12 13

Glibenclamide was initiated at 0.1 mg/kg/d and titrated to 1 mg/kg/d based on capillary glucose monitoring, according to protocols established in international studies, including those by Rafiq et al and Beltrand et al. 3 7 The infant achieved sustained euglycemia and was successfully transitioned off insulin, mirroring outcomes reported in other studies of patients with ABCC8-related PNDM. Long-term data from Bowman et al further support the durability of glycemic control and quality-of-life improvements with sulfonylurea therapy, with up to 88 to 94% of patients being able to discontinue insulin entirely. 13 Notably, studies by Sagen et al and Rafiq et al have demonstrated that targeted genetic diagnosis and individualized treatment can be effectively implemented even in low-resource settings, reinforcing the feasibility and impact of this approach in diverse clinical environments. 7 11

Even though the clinical outcome in this case was highly favorable, there remain numerous challenges, including delayed genetic test results and the lack of commercially available pediatric sulfonylurea suspension formulations, necessitating customized drug preparation. Moreover, long-term follow-up and continuous glucose monitoring were limited, leaving gaps in fully evaluating neurodevelopmental outcomes. This is crucial, as certain specific pathogenic ABCC8 variants have been reported to cause additional neurological sequelae, including epilepsy and severe developmental delays. 3 4 10

Several key lessons can be gained from the management of this case. First, a high index of suspicion for NDM should be maintained for infants under the age of 6 months presenting with unexplained hyperglycemia or ketoacidosis. Early diagnosis can improve treatment options and long-term outcomes significantly. In this patient, identification of the ABCC8 mutation allowed for early transition from insulin to oral sulfonylureas, which are both effective and more practical, especially in low-resource settings. Second, the successful use of custom-compounded glibenclamide suspension in this patient highlights how practical pharmacological adaptations can bridge resource gaps in low-income countries, without lowering the quality of therapeutic outcomes.

Additionally, the importance of a multidisciplinary approach—involving pediatricians, endocrinologists, geneticists, pharmacists, and caregivers—cannot be overstated in managing monogenic diabetes effectively. This cooperative framework lays the foundation for empowering regional health care teams, as clear communication and shared planning allow for continued delivery of high-quality care, which is essential for maintaining long-term care even in geographically underserved areas.

Ultimately, the course of events emphasizes the pivotal role of genetic diagnosis in pediatric endocrinology and demonstrates that, even in low-resource settings, personalized and targeted therapies are not only feasible but significantly effective when supported by a structured caregiver education and clinical vigilance.

## Conclusion

PNDM is a rare condition that presents diagnostic and treatment challenges in resource-limited settings. This report adds to the limited data on NDM in sub-Saharan Africa, emphasizing the potential of tailored interventions based on genetic findings. We have shown the importance of considering monogenic diabetes in neonates with hyperglycemia and how early genetic testing can guide effective, individualized therapy, which markedly improves outcomes. Lastly, we emphasize on the importance of expanding access to neonatal genetic testing and developing practical guidelines for local preparation and use of standard medications.
